# Design and validation of the scale for the adoption of artificial intelligence in the online shopping experience of Peruvian consumers

**DOI:** 10.3389/frai.2025.1712614

**Published:** 2026-01-20

**Authors:** José Joel Cruz-Tarrillo, Jose Tarrillo-Paredes, Karla Liliana Haro-Zea, Robin Alexander Díaz Díaz Saavedra

**Affiliations:** 1 Universidad Peruana Unión, Lima, Peru; 2 Universidad Autonoma de Baja California, Tecate, Mexico

**Keywords:** artificial intelligence, customer, experience, factor analysis, measurement, psychometrics, scale

## Abstract

Artificial intelligence has become a crucial tool for effective customer management; therefore, this research aims to design and validate a scale measuring the adoption of artificial intelligence in the customer experience. It is approached from a quantitative methodology perspective and an instrumental design. A survey was conducted among 528 customers who frequently make virtual purchases. Then, an exploratory analysis was conducted to determine the factor structure of the scale, followed by a confirmatory analysis to validate the construct. On the other hand, an invariance analysis was conducted to determine whether the construct varies across groups. The results show a multidimensional scale of 16 items grouped into 4 factors (trust in AI, perception of AI, knowledge of AI, shopping experience). Each factor consists of four items, using a Likert-type response scale where 1 indicates “totally disagree” and 5 indicates “totally agree”. In conclusion, the proposed scale is a valid measure. It can be used to continue exploring this concept in other latitudes, serving as a valuable tool for entrepreneurs to make an effective diagnosis of this new technology.

## Introduction

Artificial intelligence has become a revolutionary technology in the field of marketing, particularly in the management of customer experience. It has generated new ways of interacting with customers. However, despite the growing exponential increase in the use of this technology, the pending agenda on the factors that influence the adoption of AI by consumers is still an exploratory field that requires valid and reliable scales. Undoubtedly, it is crucial to comprehend how consumers utilize and engage with AI-based technologies (Martínez-Rolán et al., 2025). Within this framework, the advancement of artificial intelligence systems has a significant impact on management (Mancuso et al., 2025). The opportunities offered by artificial intelligence are among the most significant that technology has to offer, as they have the potential to add substantial value and provide a competitive advantage (Kuzior et al., 2023).

Several factors have been identified, including data capture experience, classification experience, and anthropomorphic experience (Wang et al., 2024). Likewise, the literature offers a conceptual framework that assesses collaborators’ awareness of AI using the “augmented-exhausted” model, which emphasizes people’s cognitive and emotional responses to technology (Gui et al., 2025). However, the need for scales that reflect the experiential complexities of technology use is highlighted.

On the other hand, generative AI is classified into four key areas: services, advertising, innovation, and consumer concerns (Chan and Choi, 2025). However, the success of marketing strategies will largely depend on understanding the level of AI adoption by consumers. Along the same lines, the adoption of robot-assisted technologies in the Retail sector has been explored (Shehawy et al., 2025). On the other hand, a scale has been developed to evaluate the customer experience in immersive platforms (Rahman et al., 2025), which highlights attributes such as personalization, privacy, and efficiency. Similarly, a scale focused on the interaction of hotel guests was developed (Fang et al., 2024), highlighting the need to understand aspects such as the perception of competition, closeness, and pleasant interaction —important elements in the customer experience.

Due to the growing interest in continuing to explore this field, a significant problem has been identified. There is currently no standardized scale for measuring the adoption of artificial intelligence in the field of customer experience. In response to this need, this research aims to design and validate a psychometric scale that rigorously assesses consumers’ perceptions, attitudes, and behaviors towards the incorporation of AI technologies in their interactions with commercial stores.

### Literature review

The adoption of AI in the customer experience has recently revolutionized interactions between companies and consumers, offering personalized, efficient, and automated solutions. However, it is crucial to understand the factors that influence its acceptance and use by customers for this technology to be effective. This theoretical framework presents the primary models and theories that form the conceptual basis for designing and validating the scale. Likewise, they evaluate the adoption of AI from various theoretical perspectives to integrate key concepts, such as the perception of value, trust, knowledge about AI, and the shopping experience, as measured by advanced technologies.

The UTAUT (Unified Theory of Acceptance and Use of Technology) model, proposed by Venkatesh et al. (2003), is a more compact model for explaining the adoption and use of new technologies. The model argues that four main factors influence the adoption of technology. The expectation of performance is one of the factors, it highlights the perception that technology improves performance, the second factor is the expected ease of use, maintaining that technology will be easy to use and learn, as a third factor I raise social norms, highlighting that the influence of important people in the adoption of technology and finally the facilitating conditions, encompassing external factors that facilitate or hinder the use of technology (Ayaz and Yanartaş, 2020; Lai et al., 2024). In the context of AI in the customer experience, this model enables the identification of how consumers perceive the value of AI, highlighting 3 key dimensions for this scale: trust in AI, perception of value, and knowledge of AI.

Another theory that supports this study of planned behavior (TPB) is supported by Ajzen (1991) supports three of the proposed variables, trust in AI, perception of value, and knowledge of AI. Hagger and Hamilton (2025) establishes that purchase intention is determined by three components the attitude towards use that comes to be the subjective evaluation of the positive and negative that would come to be the use of technology; the second is the subjective norms which represent the influence of social expectations on the decision to adopt the technology and the last is the perceived behavioral control, which encompasses the perception of basic skills and resources to make effective use of technology (Bosnjak et al., 2020). Regarding the use of AI in the customer experience, TBT facilitates an understanding of how personal attitudes, social, and technical knowledge influence consumers and their willingness to interact with AI-based systems.

Likewise, the SERVQUAL model, which measures the quality of service adapted to the digital context by Parasuraman et al. (2005), encompasses specific dimensions related to the interaction with advanced technology, such as reliability, responsiveness, personalization, and empathy. In the field of AI, these dimensions are directly related to the customer experience (Guillermo et al., 2025) because they enhance the accuracy of responses, attention, and personalization of interactions (Arli et al., 2024). The shopping experience, as measured in the context of AI, aligns with trust in AI according to the SERVQUAL model, supporting previous models and validating the shopping experience dimension. The customer experience model also supports the purchase experience dimension that was proposed for the design of this scale. Verhoef et al. (2009) describe the customer experience as a multidimensional, interactive, and above all, dynamic phenomenon that encompasses various points of contact between the company and the customer. Based on cognitive, emotional, and behavioral aspects, the first aspect includes the information and knowledge obtained by the customer during the interaction process, the second includes the emotions and feelings generated in that process, and the third is related to the actions and decisions that the customer makes as a result of the interaction (Zha et al., 2023). AI, on the other hand, shows a significant impact on this model of customer experience, for which Omeish et al. (2024) mentions that it improves the relevance of information, creates positive emotional experiences, and is an enabler of purchase decisions; as AI measures the shopping experience, it reflects this complex interaction that technology maintains with the customer.

On the other hand, the customer experience model supported by Kotler et al. (2020) in Marketing 4.0, the focus is on integrating digital channels, where the customer experience is managed through social, mobile, big data, and analytical networks. Likewise, the customer is viewed as a browser that interacts with brands across various digital platforms. AI is used for data analysis and automation. In Marketing 5.0, Kotler et al. (2022) reevaluate the transfer of a standardized digital experience to a hyper-personalized and highly intelligent one, where AI does not replace the human, but works as a complement, becoming a central actor in the customer experience. Additionally, it suggests that the customer experience can be managed through five key stages: attraction, interaction, conversion, loyalty, and advocacy (González-Ferriz, 2021). AI becomes a tactical tool in each of these stages, allowing it to personalize the attraction, optimize interaction actions, facilitate conversion through memorable experiences, retain customers, and generate advocacy due to exceptional satisfaction. Therefore, this theory supports and aligns with the dimensions of trust in AI, perception of value, knowledge in AI, and shopping experience.

Trust in AI is a determining element when adopting intelligent technology (Henrique and Santos, 2024; Li et al., 2024), implying the degree of security that is placed in AI systems, namely, the trust that these systems provide security, precision, transparency, benevolence, and integrity. As the criteria above are met, consumers will show confidence in adopting AI (Choung et al., 2023).

Perception in AI is based on how users interpret, evaluate its usefulness, and form attitudes towards AI systems, influenced by their previous experiences, knowledge, emotions, and beliefs (Teepapal, 2025). Similarly, Kelly et al. (2023) recognize that the usefulness of technology is related to the degree to which AI contributes to the emotional and personalized experience of the customer, rather than being framed in terms of functional efficiency.

Knowledge about AI: It is vital to mitigate the uncertainty about the adoption of new technologies such as AI, Hasan Emon and Khan (2025) understanding AI requires a fundamental grasp of what artificial intelligence is, from its operation to acknowledging its limitations.

The shopping experience measured by AI: it is based on the customer experience, which is a comprehensive construct that groups cognitive, emotional, and behavioral perceptions through the process of interaction with a brand; in the AI plane, this dimension refers to the customer’s subsequent reactions when interacting with AI functionalities, which develops capacities to listen, predict, generate, and interact (Li et al., 2025). In Marketing 5.0, Kotler et al. (2022) argue that correctly implemented AI can enhance the perceived value of the experience and make everyday or routine interactions extremely memorable.

How do people come to adopt artificial intelligence? A new model points to four linked factors: trust, perception, knowledge, and the resulting overall customer experience (2023–2025). These are not isolated elements; they constantly influence each other through psychological and social channels. Think of trust not as something fixed, but as a judgment that can change. People build this judgment by evaluating an AI’s ability, reliability, and ethical soundness, which helps them manage their natural distrust of new technologies (Gillis et al., 2024; Lalot and Bertram, 2025). Ultimately, this sense of trust is the crucial link that turns a person’s general opinion of AI into an actual willingness to use it, making automated services less intimidating.

What really matters for acceptance is how a user perceives a specific AI. Does it seem useful? Is it easy to understand? Does it seem fair, and even a little human? Studies show that designs that mimic human traits make the system seem more credible (Sfar et al., 2025). And it also works the other way around: when an AI is transparent and can explain its reasoning, it directly refines user confidence and makes the interaction seem less risky (Schnake et al., 2025). Then there is knowledge. What a user already knows about AI, whether through learning or practical use, reduces their anxiety and determines how their perception consolidates into trust (Suryadi et al., 2025). A more knowledgeable user is simply better at judging an algorithm’s performance.

The customer experience is the big picture: the holistic perception that the user gets from all their interactions with AI. This is based on aspects such as personalization, how pleasant it is, and its high efficiency. Recent studies show that when AI behaves like a human being, user satisfaction and the likelihood of purchase increase dramatically (Kumar et al., 2025). Therefore, a good experience has two effects: it deepens trust and consolidates the view of AI as a competent and beneficial partner.

**Figure 1:**
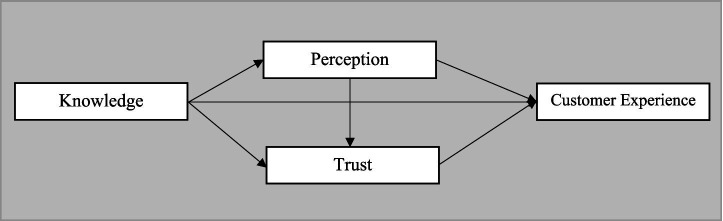
Conceptual model of research.

So how does all this fit together? The model suggests that a user’s prior knowledge and experiences directly influence both their perception and their trust. Trust operates as the central pillar that defines excellence in customer service. The user’s perspective, in turn, shapes this experience through a double effect: immediately, and by modulating the level of security that the person is willing to grant to the service. On a higher level, global considerations such as moral principles, data protection, and clarity in processes emerge. These elements constitute a contextual basis with the power to reinforce or, conversely, undermine the essential links in the model. Based on fundamental concepts about the adoption of and faith in technology, this comprehensive proposal reveals that the implementation of artificial intelligence is far from being a one-off choice. Rather, it is a constant evolution, fueled relentlessly by the dialogue between reason, sensitivity, values, and learning gained through practice (see Figure 1).

## Materials and methods

### Creation of items

This stage involved an exhaustive review of the literature in databases such as Scopus and Web of Science, resulting in the identification of 20 items. In a preliminary review conducted by the research team, four items were identified that showed conceptual redundancy, overlap in content with other items, and were not very relevant to the construct. Before beginning the formal validation procedure, it was decided to eliminate these items in order to optimize internal consistency and improve the quality of the instrument.

Then, the first version of 16 items was prepared, with a Likert-type measurement scale ranging from 1, totally disagree, to 5, totally agree. Content validation was carried out with the participation of seven marketing experts (including teachers and managers) who evaluated criteria such as adequacy, consistency, relevance, and clarity of the items. The rubric for evaluating these criteria used a scale where 1 = does not meet the criterion, 2 = low level, 3 = moderate level, and 4 = high level. After applying the Aiken coefficient formula, a value of 0.98 was obtained on the overall scale.

### Design and sample

Due to its characteristics, this research is approached from a quantitative methodological perspective, since it focuses on the collection and analysis of surveys. In addition, it employs an instrumental design aimed at developing, validating, and evaluating a measurement instrument that enables the collection of accurate and reliable information about the adoption of artificial intelligence in customer experience (Ato et al., 2013). The sample consisted of 528 customers who made purchases using virtual platforms.

The participants who took part in the study were selected using non-probability convenience sampling. This was in line with the study’s objective and the need to include customers who had made online purchases. It should be noted that in order to participate in this study, it was a requirement that participants had made an online purchase in the last 3 months and had interacted with an automated component such as chatbots or other AI-based tools. Furthermore, all participants expressed their consent to participate voluntarily.

### Data collection

To collect data, it was necessary to use the Microsoft Forms platform, through which the instrument was designed virtually, facilitating its accessibility and application. Subsequently, this questionnaire was distributed through the WhatsApp social network, enabling it to reach consumers quickly and effectively.

The geographical areas covered by the study were the city of Lima, due to its high concentration of companies with well-established customer experience management, which provides a relevant scenario for analyzing AI adoption. Likewise, in order to obtain a diverse sample, the study also collected data from the San Martin region of Peru. This heterogeneity allows for more accurate estimates and helps to generalize the results. On the other hand, these geographical areas were chosen due to the researchers’ proximity to the organizations, which facilitated coordination with the participants. Participants had to meet the criteria of having made online purchases and having interacted with artificial intelligence tools, either before, during, or after the purchase process, thus ensuring the relevance of their answers for the study.

### Data analysis

For data analysis, it was necessary to develop it in three phases. In the first phase, the evaluations provided by a group of expert judges were analyzed, which assessed the sufficiency, coherence, relevance, and clarity of each questionnaire item. To systematize this information, Aiken’s V coefficient (Penfield and Giacobbi, 2004) was used to quantify the degree of agreement among the judges on the content validity of the proposed items. In the second phase, we sought to identify the underlying structure of the construct by applying an exploratory factor analysis (EFA), mediante el método facotrización de ejes principales y la rotación varimax (Winter and Dodou, 2012; Jung and Lee, 2011) a statistical technique that allowed us to determine the grouping of the items into four latent factors. Subsequently, a confirmatory factor analysis (CFA) was conducted to verify the model’s adequacy (Graham et al., 2003). The estimation method used was maximum likelihood (Fong and Ho, 2015), and the model was evaluated using the Chi-square (*χ*^2^) absolute fit indices, RMSEA (Root Mean Square Error of Approximation) and the incremental fit indices CFI (Comparative Fit Index), TLI (Tucker-Lewis Index), NFI (Normative Fit Index), RFI (Relative Fit Index), IFI (Incremental Fit Index) (Lamoureux et al., 2007). Finally, the third phase aimed to evaluate the stability of the instrument in groups through a factorial invariance analysis, which enabled us to determine whether the scale measures the construct equivalently across different populations.

## Results

In Table 1, the distribution of gender is presented, showing a balance between the two genders, with a slight advantage for males. Likewise, a clear predominance of single consumers is observed, which implies a profile of more independent consumers with purchase decisions focused on personal interests. On the other hand, regarding academic training, its characteristics indicate that it is an informed, demanding, and rational consumer profile in their purchase decisions. Additionally, referring to the region of preference, a significant percentage of consumers are located in the jungle region. This is due to the characteristics of the study.

**Table 1:** Participants’ demographic profile (*n* = 528).

Variables	Categories	Frequency	Percentage
Sex	Male	275	52.1%
	Female	253	47.9%
Marital status	Single	444	84.1%
	Married	73	13.8%
	Divorced	8	1.5%
	Widowed	3	0.6%
Instructional level	Elementary	2	0.4%
	Secondary	56	10.6%
	University	401	75.9%
	Postgraduate	69	13.1%
Geographic location	Coast	27	5.1%
	Mountain	20	3.8%
	Jungle	481	91.1%

### Exploratory factor analysis of the scale

Table 2 presents the results of an exploratory factor analysis, with a KMO value of 0.964, a chi-square approximation of 7064.146, and a significance level of 0.000. Therefore, the data are highly suitable for performing a factor analysis. The total variance extracted is 77,820, where the four factors extracted through the principal axis factorization method with a Varimax rotation are evident. Likewise, the exploratory factor analysis revealed a structure composed of four differentiated dimensions that explain the adoption of artificial intelligence in the customer experience. The first dimension, labeled “trust in AI,” groups items with loadings ranging from 0.696 to 0.734. The second dimension, referred to as AI perception, has factor loadings ranging from 0.615 to 0.677. The third dimension, referred to as knowledge of AI, encompasses items with factor loadings ranging from 0.563 to 0.648, while the fourth dimension, labeled “shopping experience,” has factor loadings ranging from 0.516 to 0.659.

**Table 2:** Exploratory factor analysis of the data.

Instrument items	Factor			
	1	2	3	4
Confidence in AI CR = 0.920, AVE = 0.742, *α* = 0.919				
AI systems act ethically in my interactions with them.	**0.734**	0.312	0.257	0.273
The answers provided by AI tools are reliable.	**0.725**	0.292	0.279	0.323
I trust brands to protect my data by using AI technologies.	**0.696**	0.225	0.258	0.275
I feel confident in allowing AI to make automated decisions in my customer experience.	**0.696**	0.241	0.307	0.313
Perception in AI CR = 0.923, AVE = 0.751, *α* = 0.923				
AI systems save me time in my interactions with brands.	0.279	**0.677**	0.399	0.261
AI tools improve the quality of my customer experience.	0.308	**0.668**	0.339	0.264
AI personalizes my experiences in relevant and appropriate ways.	0.345	**0.654**	0.348	0.309
The AI services I use meet my needs efficiently.	0.302	**0.615**	0.439	0.318
Knowledge of AI CR = 0.840, AVE = 0.569, *α* = 0.838				
AI is a standard technology in the brands I consume.	0.274	0.284	**0.648**	0.204
I have used services that use AI, such as chatbots or virtual assistants.	0.175	0.328	**0.647**	0.255
I feel comfortable interacting with artificial intelligence-based tools.	0.322	0.401	**0.564**	0.275
I am aware of the artificial intelligence tools that the brands I interact with utilize.	0.248	0.194	**0.563**	0.142
Purchase experience CR = 0.909, AVE = 0.716, *α* = 0.908				
I prefer to interact with brands that use AI to customize my options.	0.445	0.292	0.299	**0.659**
I am satisfied with the experiences offered by services that use AI.	0.415	0.385	0.263	**0.620**
The answers created by AI tools are accurate and useful.	0.405	0.294	0.254	**0.612**
I use AI tools as a basis for comparing products or services.	0.382	0.277	0.360	**0.516**

CR: Composite Reliability; AVE: Average Variance Extracted; α: Cronbach’s alpha.

As can be seen, the factor loadings for each item exceed the threshold of 0.40, confirming the relevance of the items in their contribution to the proposal. Likewise, the sedimentation graph was analyzed, showing a clear inflection point supporting the retention of four factors. In addition to performing a parallel analysis, the cross-loadings were reviewed (see Table 2) and those most relevant to each factor were considered.

### Confirmatory factor analysis

**Figure 2:**
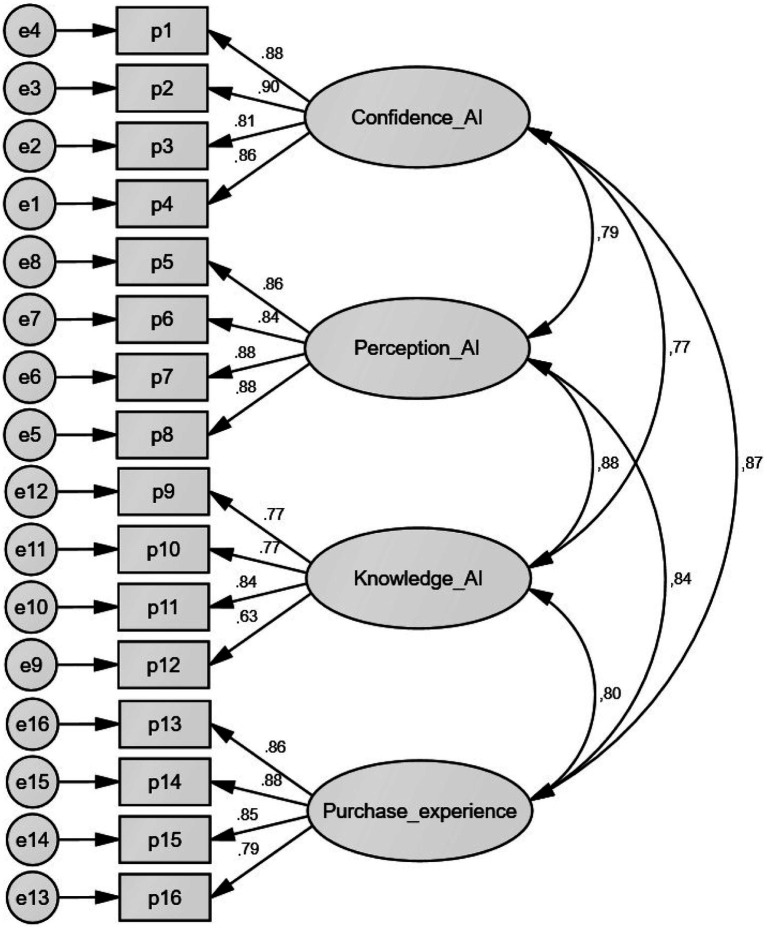
Confirmatory research model.

Figure 2, reports the scale adjustment indicators, showing an excellent adequacy of the measurement model. The chi-square value is 181.798 with 98 degrees of freedom and a *p*-value of 0.000 (*χ*^2^ = 181.798/df = 98); therefore/df = 1.855. This result is less than 5; therefore, it agrees with the established theory (Sahoo, 2019). On the other hand, the GFI index is 0.959, and the AGFI is 0.943, both of which exceed the minimum threshold of 0.80. In addition, the incremental adjustment indices. As the NFI is 0.975, the RFI is 0.969, the CFI is 0.988, the TIL is 0.985, and the IFI is 0.98, all of which exceed the recommended value of 0.90 (Hair et al., 2020). In addition, the RMSEA returned a value of 0.40, which is less than 0.08, indicating a very low approximation error. Confirmatory factor analysis is robust and supports the structural validity of the proposed instrument.

### Factor invariance analysis

Presents the invariance indices for the various models evaluated. Concerning the Chi-square (*χ*^2^), the models present *p*-values < 0.001. Likewise, the SRMR shows a progressive decrease in index (M0 = 0.028, M1 = 0.035, M2 = 0.036, M3 = 0.036), and, in addition, all values are below 0.08, indicating an excellent model fit. On the other hand, the RMSEA presents values below the critical threshold of 0.06, and the confidence intervals fall within the indicated ranges. The TLI and CFI are all well above the recommended level in the theory (0.90), indicating an important adjustment. The TLI ranges from 0.965 (M0) to 0.971 (M3), and the CFI remains between 0.971 and 0.972, indicating that all models are adequate and suggesting no gender variability in the perception of technology adoption in the customer experience.

## Discussion

As time passes, artificial intelligence becomes increasingly relevant in organizations, and the implementation and use of these technologies have evolved from being an option to a necessity. This is because consumers are becoming increasingly demanding and informed, and they require instant answers to their questions. Therefore, the objective of this research is to design and validate a scale of adoption of artificial intelligence in the customer experience. Although the customer experience indeed extends into various industries, this study focuses on the customer experience in online purchases, considering that this has a significant influence on customer satisfaction, efficiency, and competitive advantage.

AI is considered an appropriate tool for customers to improve their experience and interaction with brands (Carvalho et al., 2023; Chen and Prentice, 2025). In some organizations, there has been a significant improvement in the customer experience, perceived service quality, personalization, and brand trust, resulting in increased commitment and loyalty (Carvalho et al., 2023). AI enhances the customer experience by enabling personalized interactions, improving customer engagement through interactive agents, providing data-driven insights, and facilitating informed decision-making throughout the customer’s purchase process (Tran, 2024).

This scale has been validated among more independent consumers, with purchase decisions focused on personal interests. This type of consumer acts autonomously, guided by their own needs, preferences, and criteria, which reflects a more deliberate purchasing behavior oriented towards individual satisfaction. Likewise, concerning the level of academic training of the participants, it allows identifying a more informed, demanding, and rational consumer profile that directly influences the decision-making process.

In addition, the exploratory factor analysis demonstrates the structural validity of four dimensions suitable for measuring the adoption of artificial intelligence in the customer experience. These findings contribute to the theoretical understanding of how consumers interpret and adopt innovative technologies in their purchasing process. Likewise, the findings of the confirmatory factor analysis indicate adequate structural validity of the scale, as the chi-square test on the degrees of freedom (*χ*^2^ = 1,855, df = 98) falls within the recommended range in the literature (Sahoo, 2019), indicating an adequate fit. On the other hand, the fit indices far exceeded the threshold (CFI = 0.988, TLI = 0.985, and RMSEA = 0.040), confirming that the factorial structure consistently represents the empirical information.

On the other hand, the scale was subjected to an invariance analysis, which evidences a solidity when making a diagnosis between gender groups. The SRMR index shows a slight variation between the compared models, remaining below the critical threshold. Similarly, the RMSEA is below the critical values, which reinforces the model’s adequacy. The incremental adjustment indices, such as the TLI and CFI, are located at relevant levels, higher than the recommended cut-off points, thus confirming a high degree of adjustment. These results indicate that the scale remains stable between men and women, reinforcing the idea that both genders perceive the adoption of artificial intelligence in the customer experience in a similar way.

Although notable contributions have been made in recent times to measuring the customer experience, there are still theoretical and methodological gaps that warrant further attention. The literature has shown a growing interest in understanding how AI impacts consumer perceptions, emotions and behaviors in various industries and service scenarios, namely: a model has been proposed to evaluate the customer experience specifically in products that integrate artificial intelligence functions, pointing out that the interaction with these systems substantially modifies consumer expectations compared to traditional products (Wang et al., 2024), the quality of robotic service, highlighting the need to redefine evaluation metrics in the absence of direct human components (Prentice and Nguyen, 2021), the effect of automated social presence in AI-based services has been explored, evidencing that customers attribute social traits to virtual agents, which influences their service experience (Liao et al., 2024).

The impact of psychological anthropomorphism on the interaction with AI was also analyzed, concluding that the perception of human attributes in intelligent systems enhances the user’s emotional connection (Shen et al., 2024), empathic creativity in frontline employees within a robotic service environment was addressed, highlighting the importance of soft skills in the age of automation (Do et al., 2023). Attitudes towards artificial intelligence at work were analyzed, providing opportunities to better understand and measure workers’ attitudes towards the application of AI at work comprehensively (Park et al., 2024). In the field of tourism, the perceived intelligence of attendees with artificial intelligence for travel was analyzed(Ling et al., 2025), a scale was designed on artificial intelligence in health tourism, an expanding industry that requires high levels of trust and technological precision (Wang et al., 2022), in the educational field, attitudes towards artificial intelligence of university students were analyzed (Al-Shumrani, 2025). A framework for AI-mediated human resources development has also been proposed, emphasizing the need for adaptability in training processes (Kambur and Akar, 2022).

As shown in previous lines, despite advances in the literature, an important gap has been identified: the absence of a scale specifically designed to measure the adoption of artificial intelligence in the customer experience. This study fills this gap by proposing a scale with four dimensions that measures trust, perception, knowledge, and experience with AI. Such a scale not only enriches the academic environment but also provides organizations with a useful tool to better understand consumer behavior.

Despite the relevant findings, this research is not without limitations. First, the sample consisted of a consumer from a specific geographical and cultural context, which may limit the generalizability of the results.

## Conclusion

Based on the results obtained in this research, the following conclusions have been reached:

The factor analysis confirms the psychometric solidity of the scale, reflecting a coherent and well-defined structure focused on trust, perception, knowledge, and the consumer experience with artificial intelligence. In addition, the factors are clearly differentiated and relevant in this type of study of consumer behavior in contexts of interaction with artificial intelligence. Along the same lines, the adjustment indices obtained reflect a factorial model with excellent fit, which supports the structural validity of the proposed instrument, consolidating its usefulness in the field of marketing and customer experience measurement using artificial intelligence.

On the other hand, it is concluded that the results of the model, which is highly configurational, metric, scalar, and strict invariance, indicate that the model maintains an adequate and robust fit in all stages (M0 to M3), without deterioration in the fit indices or the differences between successive models. Additionally, the variations in the CFI and RMSEA values fall within the recommended range, confirming the instrument’s invariance. This evidence supports the notion of equality between groups, suggesting that both male and female consumers perceive the construct similarly.

## Data Availability

The original contributions presented in the study are included in the article/supplementary material, further inquiries can be directed to the corresponding author.
